# 2-[(4,6-Di­amino­pyrimidin-2-yl)sulfan­yl]-*N*-(2-methyl­phen­yl)acetamide

**DOI:** 10.1107/S1600536814015256

**Published:** 2014-07-05

**Authors:** S. Subasri, Timiri Ajay Kumar, Barji Nayan Sinha, Venkatesh Jayaprakash, Devadasan Velmurugan

**Affiliations:** a Centre of Advanced Study in Crystallography and Biophysics, University of Madras, Guindy Campus, Chennai 600 025, India; b Department of pharmaceutical Sciences, Birla Institute of Technology, Mesra, Ranchi, India

**Keywords:** crystal structure

## Abstract

In the title compound, C_13_H_15_NOS, the plane of the pyrimidine ring makes a dihedral angle of 54.73 (9)° with that of the *o*-tolyl ring. The mol­ecule adopts an extended conformation, which is evident from the C—C(=O)—N—C_ar_ (ar = aromatic) torsion angle of 178.42 (15)°. In the crystal, mol­ecules are linked *via* pairs of N—H⋯N hydrogen bonds, forming inversion dimers with an *R* ^2^_2_(8) ring motif. The dimers are linked by N—H⋯O and C—H⋯O hydrogen bonds, with the O atom accepting three such interactions, forming sheets parallel to (100).

## Related literature   

For the synthesis of the title compound, see: Xu *et al.* (2010 ▶). For the biological activity of pyrimidine derivatives, see: Hocková *et al.* (2003 ▶, 2004 ▶); Perales *et al.* (2011 ▶); Xu *et al.* (2010 ▶).
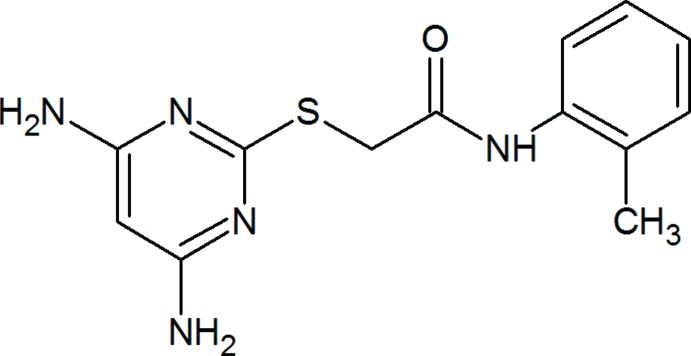


## Experimental   

### 

#### Crystal data  

C_13_H_15_N_5_OS
*M* *_r_* = 289.36
Monoclinic, 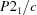

*a* = 22.782 (5) Å
*b* = 7.144 (5) Å
*c* = 8.857 (5) Å
β = 100.189 (5)°
*V* = 1418.8 (13) Å^3^
*Z* = 4
Mo *K*α radiation
μ = 0.23 mm^−1^
*T* = 293 K
0.30 × 0.25 × 0.20 mm

#### Data collection  

Bruker SMART APEXII area-detector diffractometer
Absorption correction: multi-scan (*SADABS*; Bruker, 2008 ▶) *T*_min_ = 0.687, *T*_max_ = 0.746
12825 measured reflections
3417 independent reflections
2654 reflections with *I* > 2σ(*I*)
*R*_int_ = 0.025

#### Refinement  

*R*[*F* ^2^ > 2σ(*F* ^2^)] = 0.039
*wR*(*F* ^2^) = 0.128
*S* = 1.02
3417 reflections
182 parameters
H-atom parameters constrained
Δρ_max_ = 0.25 e Å^−3^
Δρ_min_ = −0.23 e Å^−3^

### 

Data collection: *APEX2* (Bruker, 2008 ▶); cell refinement: *SAINT* (Bruker, 2008 ▶); data reduction: *SAINT*; program(s) used to solve structure: *SHELXS97* (Sheldrick, 2008 ▶); program(s) used to refine structure: *SHELXL97* (Sheldrick, 2008 ▶); molecular graphics: *ORTEP-3 for Windows* (Farrugia, 2012 ▶); software used to prepare material for publication: *SHELXL97* and *PLATON* (Spek, 2009 ▶).

## Supplementary Material

Crystal structure: contains datablock(s) global, I. DOI: 10.1107/S1600536814015256/su2743sup1.cif

Structure factors: contains datablock(s) I. DOI: 10.1107/S1600536814015256/su2743Isup2.hkl

Supporting information file. DOI: 10.1107/S1600536814015256/su2743Isup3.cml

CCDC reference: 1010947

Additional supporting information:  crystallographic information; 3D view; checkCIF report

## Figures and Tables

**Table 1:** Hydrogen-bond geometry (Å, °)

*D*—H⋯*A*	*D*—H	H⋯*A*	*D*⋯*A*	*D*—H⋯*A*
N4—H4*A*⋯N1^i^	0.86	2.26	3.115 (2)	175
N3—H3*A*⋯O1^ii^	0.86	2.27	3.045 (2)	150
N5—H5⋯O1^ii^	0.86	2.54	3.264 (3)	142
C13—H13*A*⋯O1^ii^	0.96	2.55	3.385 (3)	145

Symmetry codes: (i) 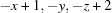; (ii) 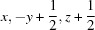.

**Fig. 1.**
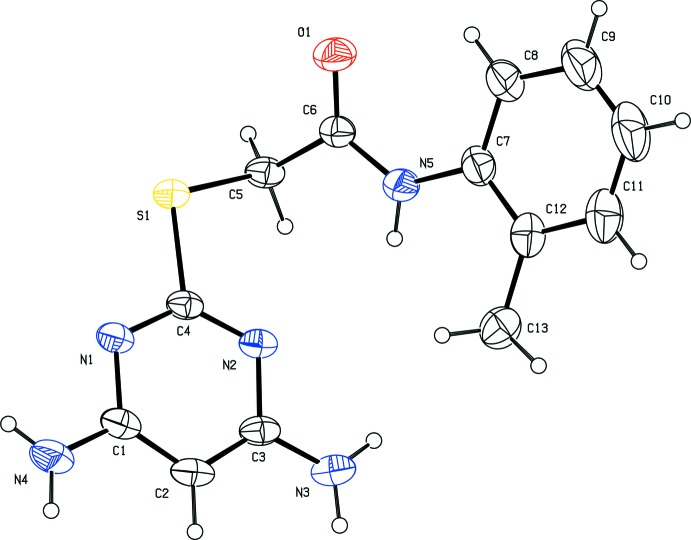
The molecular structure of the title molecule, with atom labelling. Displacement ellipsoids are drawn at the 30% probability level.

**Fig. 2.**
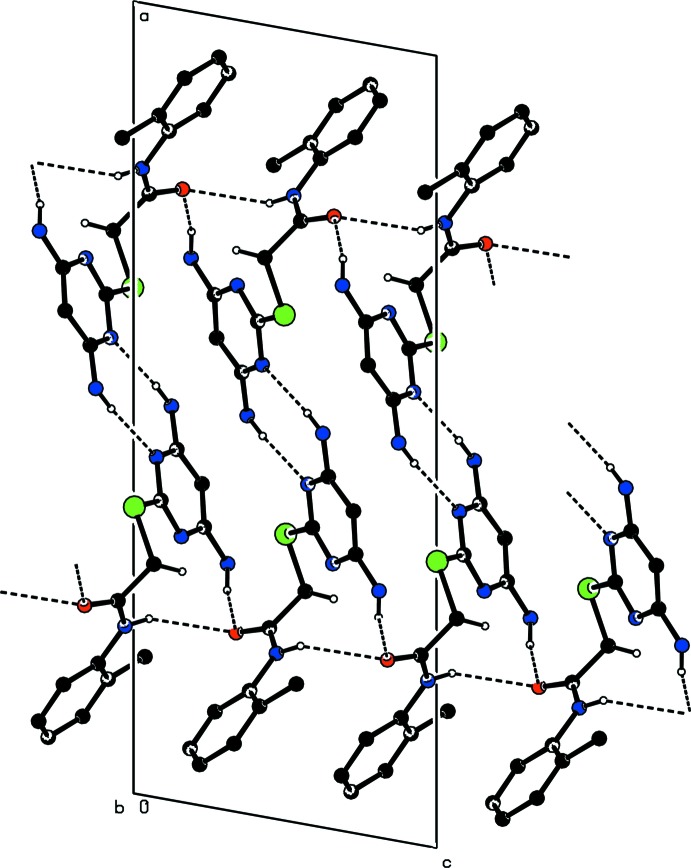
The crystal packing of the title compound, viewed along the b axis. The hydrogen bonds are shown as dashed lines (see Table 1 for details; the C and N-bound H-atoms not involved in hydrogen bonding have been omitted for clarity).

## supplementary crystallographic information

### S1. Comment

Diaminopyrimidines are an important class of six membered heterocyclic compounds with many applications. For example, some derivatives are been reported to have anticancer activity, selectively inhibiting c-Fms kinase of M-CSF-dependent myeloid leukemia cells (Xu *et al.*, 2010). Some 2,4-diamino-pyrimidine derivatives have been shown to have anti-retro viral activity (Hocková *et al.*, 2003,2004), and anti-trypanosoma brucei activity (Perales *et al.*, 2011). In search for antiviral agents the title compound was designed and synthesized for targeting NS2B-NS3 protease. We report herein on its synthesis and crystal structure.

In the title compound, Fig. 1, the pyrimidine ring (N1/N2/C1-C4) makes a dihedral angle of 54.73 (9)° with the benzene ring (C7-C12). The molecule adopts an extended conformation which is evident from torsion angle C5—C6—N5—C7 = 178.42 (15) °. The amine group, atom N3, deviates from the pyrimidine ring by -0.0672 (15) Å, while atom N4 atom deviates from the same ring by 0.0824 (18) Å. The methyl carbon atom C13 deviates from the benzene ring to which it is attached by 0.0204 (22) Å.

In the crystal, molecules are linked by pairs of N—H···N hydrogen bonds forming inversion dimers and enclosing R_2_^2^(8) ring motifs (Table 1 and Fig. 2). The dimers are linked via trifurcated N—H···O and C—H···O hydrogen bonds involving atom O1 as an acceptor (Table 1 and Fig. 2) forming forming sheets parallel to (100).

### S2. Experimental

The title compound was synthesized according to the reported procedure (Xu *et al.*, 2010). To a solution of 4,6-diamino-pyrimidine-2-thiol (0.5 g; 3.52 mmol) in 25 ml of ethanol was added potassium hydroxide(0.2g; 3.52 mmol) and the mixture was refluxed for 30 mins. Then 3.52 mmol of 2-chloro-N-phenylacetamide was added and the mixture refluxed for 1-4 h. At the end of the reaction, monitored by TLC, ethanol was evaporated in vacuo and cold water was added. The precipitate formed was filtered and dried to give the title compound as a crystalline powder (Yield of 88-96%). Block-like colourless crystals were obtained by slow evaporation of a solution in methanol at room temperature.

### S3. Refinement

The NH and C-bound H atoms were placed in idealized positions and refined using a riding model: N-H = 0.86 Å, C-H = 0.93, 0.97 and 0.96 Å for CH, CH_2_ and CH_3_ H atoms, respectively, with U_iso_(H) = 1.5U_eq_(C-methyl) and = 1.2U_eq_(N,C) for other H atoms.

### Crystal data

**Table:**

C_13_H_15_N_5_OS	*Z* = 4
*M**_r_* = 289.36	*F*(000) = 608
Monoclinic, *P*2_1_/*c*	*D*_x_ = 1.355 Mg m^−^^3^
Hall symbol: -P 2ybc	Mo *K*α radiation, λ = 0.71073 Å
*a* = 22.782 (5) Å	µ = 0.23 mm^−^^1^
*b* = 7.144 (5) Å	*T* = 293 K
*c* = 8.857 (5) Å	Block, colourless
β = 100.189 (5)°	0.30 × 0.25 × 0.20 mm
*V* = 1418.8 (13) Å^3^	

### Data collection

**Table:**

Bruker SMART APEXII area-detector diffractometer	3417 independent reflections
Radiation source: fine-focus sealed tube	2654 reflections with *I* > 2σ(*I*)
Graphite monochromator	*R*_int_ = 0.025
ω and φ scans	θ_max_ = 28.4°, θ_min_ = 1.8°
Absorption correction: multi-scan (*SADABS*; Bruker, 2008)	*h* = −30→30
*T*_min_ = 0.687, *T*_max_ = 0.746	*k* = −9→8
12825 measured reflections	*l* = −11→11

### Refinement

**Table:**

Refinement on *F*^2^	Primary atom site location: structure-invariant direct methods
Least-squares matrix: full	Secondary atom site location: difference Fourier map
*R*[*F*^2^ > 2σ(*F*^2^)] = 0.039	Hydrogen site location: inferred from neighbouring sites
*wR*(*F*^2^) = 0.128	H-atom parameters constrained
*S* = 1.02	*w* = 1/[σ^2^(*F*_o_^2^) + (0.0743*P*)^2^ + 0.1678*P*] where *P* = (*F*_o_^2^ + 2*F*_c_^2^)/3
3417 reflections	(Δ/σ)_max_ = 0.001
182 parameters	Δρ_max_ = 0.25 e Å^−^^3^
0 restraints	Δρ_min_ = −0.23 e Å^−^^3^

### Special details

**Table:**

Geometry. All esds (except the esd in the dihedral angle between two l.s. planes) are estimated using the full covariance matrix. The cell esds are taken into account individually in the estimation of esds in distances, angles and torsion angles; correlations between esds in cell parameters are only used when they are defined by crystal symmetry. An approximate (isotropic) treatment of cell esds is used for estimating esds involving l.s. planes.
Refinement. Refinement of F^2^ against ALL reflections. The weighted R-factor wR and goodness of fit S are based on F^2^, conventional R-factors R are based on F, with F set to zero for negative F^2^. The threshold expression of F^2^ > 2sigma(F^2^) is used only for calculating R-factors(gt) etc. and is not relevant to the choice of reflections for refinement. R-factors based on F^2^ are statistically about twice as large as those based on F, and R- factors based on ALL data will be even larger.

### Fractional atomic coordinates and isotropic or equivalent isotropic displacement parameters (Å^2^)

**Table:**

	*x*	*y*	*z*	*U*_iso_*/*U*_eq_	
C1	0.44402 (7)	−0.1657 (2)	1.14271 (18)	0.0541 (4)	
C2	0.40616 (8)	−0.2557 (2)	1.22577 (18)	0.0565 (4)	
H2	0.4169	−0.3687	1.2751	0.068*	
C3	0.35231 (7)	−0.1729 (2)	1.23308 (15)	0.0479 (4)	
C4	0.37612 (6)	0.06758 (19)	1.08993 (15)	0.0428 (3)	
C5	0.29403 (7)	0.3577 (2)	1.06689 (17)	0.0498 (4)	
H5A	0.2910	0.2907	1.1604	0.060*	
H5B	0.2958	0.4905	1.0902	0.060*	
C6	0.23955 (7)	0.3184 (2)	0.94817 (15)	0.0450 (3)	
C7	0.15591 (7)	0.0929 (3)	0.88301 (19)	0.0600 (4)	
C8	0.12155 (9)	0.1991 (4)	0.7684 (3)	0.0836 (7)	
H8	0.1335	0.3192	0.7471	0.100*	
C9	0.06952 (10)	0.1246 (5)	0.6865 (3)	0.1038 (9)	
H9	0.0467	0.1945	0.6089	0.125*	
C10	0.05148 (10)	−0.0489 (5)	0.7182 (3)	0.1083 (9)	
H10	0.0161	−0.0974	0.6636	0.130*	
C11	0.08558 (10)	−0.1541 (4)	0.8317 (3)	0.0879 (7)	
H11	0.0727	−0.2734	0.8524	0.105*	
C12	0.13842 (7)	−0.0871 (3)	0.9157 (2)	0.0623 (5)	
C13	0.17501 (10)	−0.2061 (3)	1.0374 (2)	0.0705 (5)	
H13A	0.1746	−0.1520	1.1364	0.106*	
H13B	0.2153	−0.2122	1.0196	0.106*	
H13C	0.1585	−0.3300	1.0339	0.106*	
N1	0.42970 (6)	0.00314 (17)	1.07572 (14)	0.0492 (3)	
N2	0.33525 (5)	−0.01003 (16)	1.15794 (12)	0.0447 (3)	
N3	0.31124 (7)	−0.2447 (2)	1.31032 (16)	0.0624 (4)	
H3A	0.2779	−0.1877	1.3088	0.075*	
H3B	0.3185	−0.3474	1.3609	0.075*	
N4	0.49728 (8)	−0.2346 (3)	1.1248 (2)	0.0820 (5)	
H4A	0.5195	−0.1723	1.0738	0.098*	
H4B	0.5091	−0.3410	1.1643	0.098*	
N5	0.20923 (6)	0.1643 (2)	0.97155 (15)	0.0572 (4)	
H5	0.2240	0.0995	1.0511	0.069*	
O1	0.22618 (6)	0.42293 (18)	0.83847 (13)	0.0687 (4)	
S1	0.361751 (18)	0.28855 (6)	1.00258 (5)	0.05785 (16)	

### Atomic displacement parameters (Å^2^)

**Table:**

	*U*^11^	*U*^22^	*U*^33^	*U*^12^	*U*^13^	*U*^23^
C1	0.0596 (9)	0.0472 (9)	0.0498 (8)	0.0097 (7)	−0.0054 (7)	0.0037 (7)
C2	0.0738 (11)	0.0425 (8)	0.0486 (8)	0.0117 (8)	−0.0017 (7)	0.0092 (6)
C3	0.0688 (10)	0.0384 (7)	0.0326 (6)	0.0011 (7)	−0.0014 (6)	−0.0002 (5)
C4	0.0536 (8)	0.0356 (7)	0.0345 (6)	0.0012 (6)	−0.0054 (5)	−0.0015 (5)
C5	0.0628 (9)	0.0354 (7)	0.0490 (8)	0.0060 (7)	0.0039 (6)	−0.0007 (6)
C6	0.0508 (8)	0.0444 (8)	0.0414 (7)	0.0090 (6)	0.0124 (6)	0.0012 (6)
C7	0.0461 (8)	0.0792 (12)	0.0535 (9)	−0.0016 (8)	0.0058 (7)	0.0023 (8)
C8	0.0543 (11)	0.1116 (18)	0.0796 (13)	−0.0008 (11)	−0.0025 (9)	0.0239 (12)
C9	0.0571 (12)	0.160 (3)	0.0862 (15)	−0.0027 (16)	−0.0096 (10)	0.0175 (17)
C10	0.0581 (13)	0.165 (3)	0.0938 (17)	−0.0216 (16)	−0.0090 (11)	−0.0151 (18)
C11	0.0645 (12)	0.1092 (18)	0.0881 (14)	−0.0245 (12)	0.0087 (11)	−0.0199 (14)
C12	0.0539 (9)	0.0765 (13)	0.0579 (9)	−0.0093 (9)	0.0139 (7)	−0.0114 (8)
C13	0.0777 (13)	0.0576 (11)	0.0757 (12)	−0.0109 (9)	0.0124 (10)	−0.0059 (9)
N1	0.0530 (7)	0.0419 (7)	0.0487 (7)	0.0059 (6)	−0.0023 (5)	0.0030 (5)
N2	0.0585 (7)	0.0362 (6)	0.0365 (6)	0.0039 (5)	0.0002 (5)	0.0000 (5)
N3	0.0830 (11)	0.0503 (8)	0.0549 (8)	0.0077 (7)	0.0146 (7)	0.0147 (6)
N4	0.0690 (11)	0.0739 (11)	0.1029 (14)	0.0298 (9)	0.0151 (9)	0.0318 (10)
N5	0.0551 (8)	0.0603 (8)	0.0521 (7)	−0.0044 (6)	−0.0020 (6)	0.0133 (6)
O1	0.0780 (8)	0.0694 (8)	0.0540 (7)	−0.0050 (6)	−0.0013 (6)	0.0188 (6)
S1	0.0529 (3)	0.0455 (3)	0.0740 (3)	0.00657 (17)	0.00824 (19)	0.02046 (18)

### Geometric parameters (Å, º)

**Table:**

C1—N4	1.345 (2)	C7—N5	1.419 (2)
C1—N1	1.358 (2)	C8—C9	1.382 (3)
C1—C2	1.387 (3)	C8—H8	0.9300
C2—C3	1.374 (2)	C9—C10	1.351 (4)
C2—H2	0.9300	C9—H9	0.9300
C3—N3	1.354 (2)	C10—C11	1.379 (4)
C3—N2	1.3625 (19)	C10—H10	0.9300
C4—N2	1.317 (2)	C11—C12	1.384 (3)
C4—N1	1.3312 (19)	C11—H11	0.9300
C4—S1	1.7630 (17)	C12—C13	1.504 (3)
C5—C6	1.505 (2)	C13—H13A	0.9600
C5—S1	1.8054 (17)	C13—H13B	0.9600
C5—H5A	0.9700	C13—H13C	0.9600
C5—H5B	0.9700	N3—H3A	0.8600
C6—O1	1.2207 (18)	N3—H3B	0.8600
C6—N5	1.335 (2)	N4—H4A	0.8600
C7—C8	1.392 (3)	N4—H4B	0.8600
C7—C12	1.392 (3)	N5—H5	0.8600
			
N4—C1—N1	115.18 (16)	C8—C9—H9	119.7
N4—C1—C2	123.50 (16)	C9—C10—C11	119.9 (2)
N1—C1—C2	121.30 (15)	C9—C10—H10	120.0
C3—C2—C1	118.03 (15)	C11—C10—H10	120.0
C3—C2—H2	121.0	C10—C11—C12	121.9 (3)
C1—C2—H2	121.0	C10—C11—H11	119.1
N3—C3—N2	114.04 (15)	C12—C11—H11	119.1
N3—C3—C2	124.29 (14)	C11—C12—C7	117.5 (2)
N2—C3—C2	121.65 (15)	C11—C12—C13	120.6 (2)
N2—C4—N1	129.18 (13)	C7—C12—C13	121.89 (16)
N2—C4—S1	119.08 (11)	C12—C13—H13A	109.5
N1—C4—S1	111.74 (11)	C12—C13—H13B	109.5
C6—C5—S1	111.95 (10)	H13A—C13—H13B	109.5
C6—C5—H5A	109.2	C12—C13—H13C	109.5
S1—C5—H5A	109.2	H13A—C13—H13C	109.5
C6—C5—H5B	109.2	H13B—C13—H13C	109.5
S1—C5—H5B	109.2	C4—N1—C1	114.78 (14)
H5A—C5—H5B	107.9	C4—N2—C3	114.77 (13)
O1—C6—N5	124.40 (15)	C3—N3—H3A	120.0
O1—C6—C5	120.05 (14)	C3—N3—H3B	120.0
N5—C6—C5	115.54 (13)	H3A—N3—H3B	120.0
C8—C7—C12	120.68 (18)	C1—N4—H4A	120.0
C8—C7—N5	121.56 (19)	C1—N4—H4B	120.0
C12—C7—N5	117.76 (15)	H4A—N4—H4B	120.0
C9—C8—C7	119.5 (2)	C6—N5—C7	128.79 (14)
C9—C8—H8	120.2	C6—N5—H5	115.6
C7—C8—H8	120.2	C7—N5—H5	115.6
C10—C9—C8	120.6 (2)	C4—S1—C5	102.08 (8)
C10—C9—H9	119.7		
			
N4—C1—C2—C3	−178.67 (16)	N5—C7—C12—C13	−1.6 (3)
N1—C1—C2—C3	2.8 (2)	N2—C4—N1—C1	−0.8 (2)
C1—C2—C3—N3	−179.76 (15)	S1—C4—N1—C1	179.04 (11)
C1—C2—C3—N2	1.9 (2)	N4—C1—N1—C4	177.99 (14)
S1—C5—C6—O1	−78.22 (17)	C2—C1—N1—C4	−3.3 (2)
S1—C5—C6—N5	100.79 (14)	N1—C4—N2—C3	5.1 (2)
C12—C7—C8—C9	0.1 (3)	S1—C4—N2—C3	−174.73 (9)
N5—C7—C8—C9	−179.1 (2)	N3—C3—N2—C4	176.08 (12)
C7—C8—C9—C10	0.9 (4)	C2—C3—N2—C4	−5.39 (19)
C8—C9—C10—C11	−0.9 (5)	O1—C6—N5—C7	−2.6 (3)
C9—C10—C11—C12	0.0 (4)	C5—C6—N5—C7	178.42 (15)
C10—C11—C12—C7	0.9 (3)	C8—C7—N5—C6	−11.7 (3)
C10—C11—C12—C13	−179.2 (2)	C12—C7—N5—C6	169.09 (16)
C8—C7—C12—C11	−0.9 (3)	N2—C4—S1—C5	9.30 (12)
N5—C7—C12—C11	178.33 (17)	N1—C4—S1—C5	−170.54 (10)
C8—C7—C12—C13	179.20 (19)	C6—C5—S1—C4	−98.07 (12)

### Hydrogen-bond geometry (Å, º)

**Table:**

*D*—H···*A*	*D*—H	H···*A*	*D*···*A*	*D*—H···*A*
N4—H4*A*···N1^i^	0.86	2.26	3.115 (2)	175
N3—H3*A*···O1^ii^	0.86	2.27	3.045 (2)	150
N5—H5···O1^ii^	0.86	2.54	3.264 (3)	142
C13—H13*A*···O1^ii^	0.96	2.55	3.385 (3)	145

Symmetry codes: (i) −*x*+1, −*y*, −*z*+2; (ii) *x*, −*y*+1/2, *z*+1/2.
